# Sub-segmental quantification of single (stress)-pass perfusion CMR improves the diagnostic accuracy for detection of obstructive coronary artery disease

**DOI:** 10.1186/s12968-020-0600-1

**Published:** 2020-02-06

**Authors:** Melanie T. P. Le, Niloufar Zarinabad, Tommaso D’Angelo, Ibnul Mia, Robert Heinke, Thomas J. Vogl, Andreas Zeiher, Eike Nagel, Valentina O. Puntmann

**Affiliations:** 1 Institute for Experimental and Translational Cardiovascular Imaging, University Hospital Frankfurt, Theodor-Stern Kai 7, 60590 Frankfurt am Main, Germany; 2 Department of Biomedical Sciences and Morphological and Functional Imaging, G. Martino University Hospital Messina, Via Consolare Valeria 1, Messina, 98100 Italy; 3 Department of Radiology, University Hospital Frankfurt, Theodor-Stern Kai 7, Frankfurt am Main, Germany; 4 Department of Cardiology, University Hospital Frankfurt, Theodor-Stern Kai 7, Frankfurt am Main, Germany

**Keywords:** Cardiovascular magnetic resonance, Myocardial perfusion, 16 segment AHA model, Sub-segmentation, Myocardial segmentation

## Abstract

**Background:**

Myocardial perfusion with cardiovascular magnetic resonance (CMR) imaging is an established diagnostic test for evaluation of myocardial ischaemia. For quantification purposes, the 16 segment American Heart Association (AHA) model poses limitations in terms of extracting relevant information on the extent/severity of ischaemia as perfusion deficits will not always fall within an individual segment, which reduces its diagnostic value, and makes an accurate assessment of outcome data or a result comparison across various studies difficult. We hypothesised that division of the myocardial segments into epi- and endocardial layers and a further circumferential subdivision, resulting in a total of 96 segments, would improve the accuracy of detecting myocardial hypoperfusion. Higher (sub-)subsegmental recording of perfusion abnormalities, which are defined relatively to the normal reference using the subsegment with the highest value, may improve the spatial encoding of myocardial blood flow, based on a single stress perfusion acquisition.

**Objective:**

A proof of concept comparison study of subsegmentation approaches based on transmural segments (16 AHA and 48 segments) vs. subdivision into epi- and endocardial (32) subsegments vs. further circumferential subdivision into 96 (sub-)subsegments for diagnostic accuracy against invasively defined obstructive coronary artery disease (CAD).

**Methods:**

Thirty patients with obstructive CAD and 20 healthy controls underwent perfusion stress CMR imaging at 3 T during maximal adenosine vasodilation and a dual bolus injection of 0.1 mmol/kg gadobutrol. Using Fermi deconvolution for blood flow estimation, (sub-)subsegmental values were expressed relative to the (sub-)subsegment with the highest flow. In addition, endo−/epicardial flow ratios were calculated based on 32 and 96 (sub-)subsegments. A receiver operating characteristics (ROC) curve analysis was performed to compare the diagnostic performance of discrimination between patients with CAD and healthy controls. Observer reproducibility was assessed using Bland-Altman approaches.

**Results:**

Subdivision into more and smaller segments revealed greater accuracy for #32, #48 and # 96 compared to the standard #16 approach (area under the curve (AUC): 0.937, 0.973 and 0.993 vs 0.820, *p* < 0.05). The #96-based endo−/epicardial ratio was superior to the #32 endo−/epicardial ratio (AUC 0.979, vs. 0.932, *p* < 0.05). Measurements for the #16 model showed marginally better reproducibility compared to #32, #48 and #96 (mean difference ± standard deviation: 2.0 ± 3.6 vs. 2.3 ± 4.0 vs 2.5 ± 4.4 vs. 4.1 ± 5.6).

**Conclusions:**

Subsegmentation of the myocardium improves diagnostic accuracy and facilitates an objective cut-off-based description of hypoperfusion, and facilitates an objective description of hypoperfusion, including the extent and severity of myocardial ischaemia. Quantification based on a single (stress-only) pass reduces the overall amount of gadolinium contrast agent required and the length of the overall diagnostic study.

## Introduction

Coronary artery disease (CAD) is a global epidemic with an increasing impact on healthcare systems [1]. Significant advances in both diagnosing and treating acute epicardial CAD have improved survival and reduced morbidity during the last decades [2]. One of the main unresolved issues in diagnosis of chronic CAD represents the definition of clinically relevant ischaemia [3, 4]. Myocardial perfusion based on cardiovascular magnetic resonance (CMR) imaging provides excellent diagnostic accuracy and prognostic value (summarised in [4]), and is an established diagnostic method in clinical practice [1, 5] In clinical practice, perfusion stress CMR is analysed and interpreted based on visually perceptible differences in peaks of contrast signal intensity and contrast kinetics [6]. Clinical reports usually summarise the extent and transmurality of hypoperfusion, based on the American Heart Association (AHA) 16 segment left ventricle (LV) model [7], as well as localisation in terms of coronary perfusion territory. Experience reveals that visual analysis of perfusion stress CMR, using the 16 segment model, can be difficult to standardise and to record accurately and reproducibly, as perfusion defects frequently involve several adjacent segments, which are often only partially involved [8, 9]. Some improvement has been achieved by the subdivision of 16 segments into 32 epi- and endocardial subsegments [5, 10]. Furthermore, as fully automated analyses, based on voxel-wise quantification, become feasible [11–14], further subdivision may be possible, improving the overall measurement accuracy of regional distribution of myocardial blood flow. However, despite the huge potential, quantitative outputs of voxel-based analyses are reported as an average of all voxel-based measurements, expressed per each transmural segment within the 16 segment model (or one of 32 subsegments, respectively). Consequently, the potential information of voxel-based measurements of spatial differences of myocardial flow is discarded, leading to several obvious problems. Firstly, mixing signals from multiple voxel signals may lead to overestimation of reduced perfusion in segments which are only partially involved. Consequently, the overestimation leads to the underestimation of peak perfusion in normal areas with high inflow of contrast agent and increase in signal intensities. This results in lower effective difference between normal and abnormal perfusion, potentially reducing overall diagnostic performance. Secondly, classifying perfusion defects, in line with presumed coronary artery distributions, may contribute inaccuracies, especially along the border territories in databases with rigid allocation of segments. Meaningful and robust ways of recording and communicating quantification results of myocardial perfusion may be useful to harness the potential of fully automated analyses and to develop reliable diagnostic matrices for artificial intelligence machine learning approaches. We hypothesise that a subdivision of the classical 16 segment model into 32 subsegments (epicardial and endocardial), 48 subsegments (circular division of the 16 segments into 3 segments each) and 96 (sub)-subsegments (dividing the 48 subsegments into epi- and endocardial) would improve the accuracy of myocardial perfusion measurement. Quantitative analysis based on LV segmentation into 96 (sub-)subsegments, each representing approximately 1% of myocardium, may considerably simplify the reporting scheme for the extent of myocardial ischaemia, laying the base for a detailed and robust reporting of voxel-wise analyses for clinical interpretation and databasing. To test this hypothesis, we undertook a proof of concept comparison study of the diagnostic accuracies of myocardial segmentation approaches (transmural 16 and 48 segments, vs. 32 vs. 96 epi- and endocardial subsegments) and based quantitative analysis of stress myocardial perfusion in patients with obstructive CAD and healthy controls.

## Methods

This is a sub-study of the prospective longitudinal, observational, and investigator-led study of T1-mapping in adult patients undergoing clinically indicated CMR examination (International T1-CMR Outcome Study NCT03749343) [15, 16]. The study protocol was reviewed and approved by the respective institutional ethics committees and written informed consent was obtained from all participants. All procedures were carried out in accordance with the Declaration of Helsinki (2013). Consecutive subjects, with typical symptoms of angina (Canadian Class Symptoms 2–3) and either a positive exercise tolerance test or more than two cardiovascular risk factors, were screened for inclusion between March 2016 and October 2017. Of these, datasets of 30 patients with obstructive CAD, defined as a stenosis of a proximal or medial vessel of ≥2 mm diameter with ≥80% diameter stenosis or 60–80% diameter stenosis and fractional flow reserve (FFR) of < 0.8, were identified for quantitative analysis [5, 17–19].

**Fig. 1:**
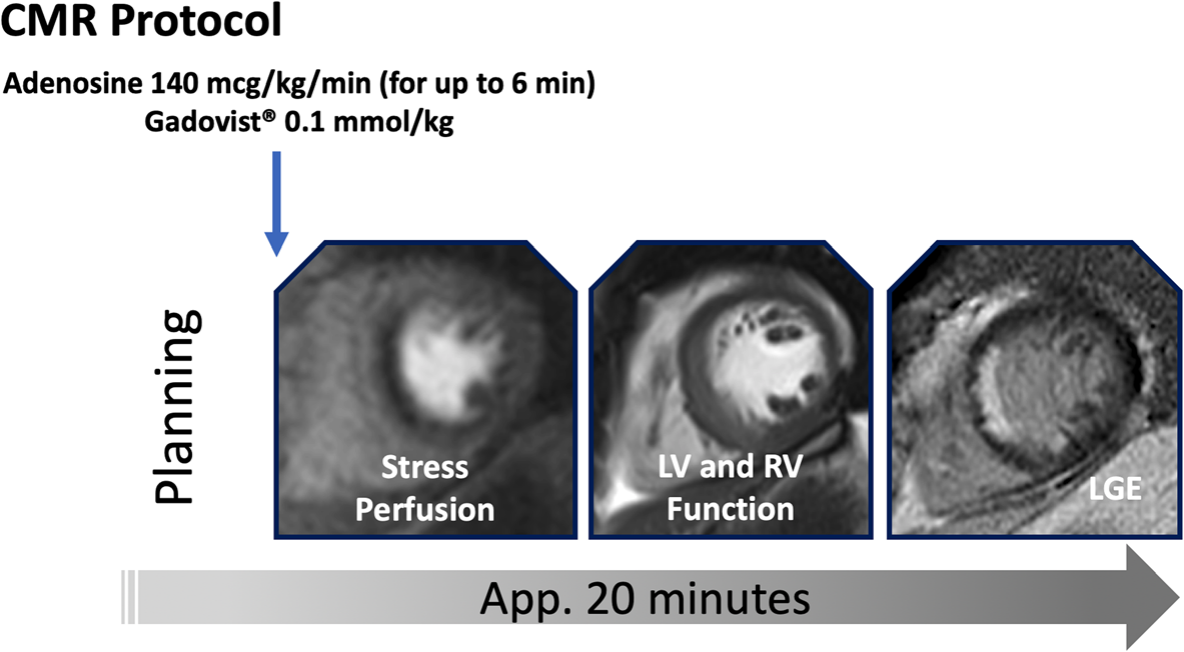
Imaging Protocol. Stress perfusion during adenosine infusion, followed by a cine imaging short axis stack and late gadolinium enhancement (LGE). The total dose of gadolinium based contrast agent (GBCA) is Gadovist® 0.1 mmol/kg body weight. Scan time for this protocol amounts to approximately 20 min

Normotensive age-gender matched healthy subjects (*n* = 20), who had a low pre-test-likelihood of CAD, did not take any regular medications, had normal routine blood tests, urine samples and CMR findings, including normal LV mass indices, served as controls. Exclusion criteria were the generally accepted contraindications to CMR, atrial fibrillation or prior coronary artery bypass surgery. All subjects underwent a routine clinical scan protocol for myocardial stress perfusion and a scar imaging, using a 3-T clinical scanner (Skyra, Siemens Healthineers, Erlangen, Germany) [20]**.** Myocardial perfusion imaging was acquired during maximal vasodilation, using continuous adenosine infusion starting at 140 μg/kg body weight/min. Dynamic image acquisition was performed during the delivery of gadolinium-based contrast agent (GBCA; gadobutrol 0.1 mmol/kg, Gadovist®, Bayer, Berlin, Germany) at 4 ml/s by an injector pump for stress, using a dual bolus delivery scheme (the first bolus with 5%, the second bolus with 100% GBCA, both followed by chaser of 20 ml saline, with a break of 30s between two boluses) [14, 21]. The 3 short axis slices, located within the middle of each 1/3 of the LV cavity (Fig. 1), planned at the end-diastole were acquired at every heartbeat [22] (Steady-state free precession sequence, TE/TR/flip-angle 2.0/3.5/35–50°, saturation preparation pre-pulse, 100 ms pre-pulse delay, typical acquired resolution of 2.5 × 2.5 × 8 mm) during the first pass of the pre- and the main bolus. Slice location was determined at 25, 50 and 75% of end-systolic LV length in the 4-chamber view. Cine images were acquired after stress perfusion, followed by late gadolinium enhancement imaging (LGE) at approximately 15 min after GBCA administration, using a mid-diastolic inversion prepared 2-dimensional gradient echo sequence (TE/TR/flip-angle 2.0 ms/3.4 ms/25°, acquired voxel size 1.4 × 1.4x8mm) with an individually adapted pre-pulse delay achieving optimally nulled myocardium. The exam cards employed are available online [22].

**Fig. 2:**
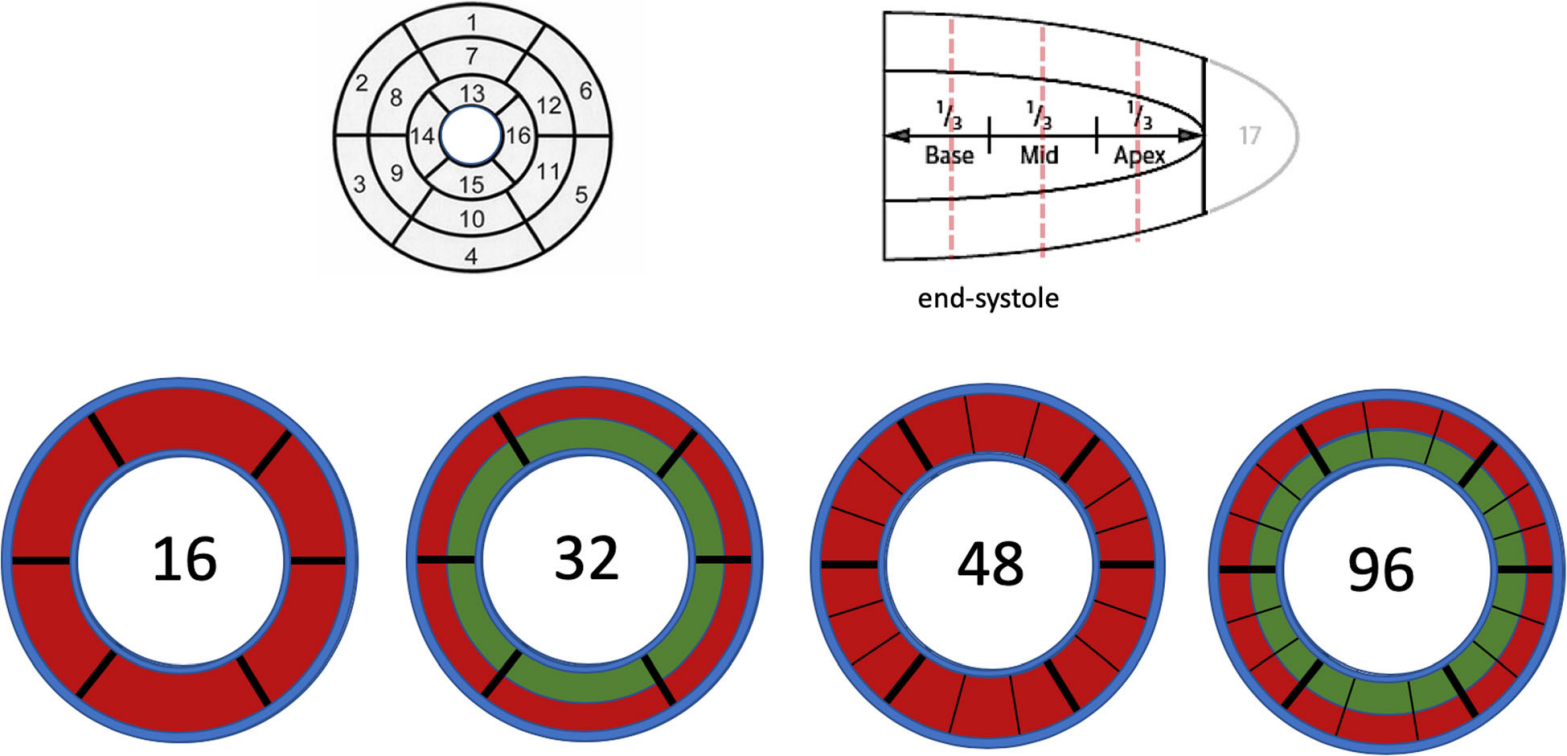
Cardiac segmentation. 16 segment model using the classic AHA model (top row and left), subdivision into 32 segments by epi- and endocardial division as well as (sub-)subdivision into 96 segments by dividing each subsegment into 3 further circumferential segments (lower row, only the mid-slice is shown for demonstration)

Postprocessing analyses were performed by non-clinical members of the core-lab team, working on anonymised datasets with no access to clinical background information. The myocardium was delineated manually, with a visual check of placement and a manual adjustment of contours to reduce inclusion of areas of blood, black rim artefacts, or pericardial fat, using MEDIS® (Leiden, The Netherlands). Then, segmentations were set automatically, using a spoke-wheel approach. The 16 AHA segments were defined as previously described and refined [9, 22]. Then, these 16 transmural segments were subdivided into epi- and endocardial subsegments using standardised inclusion of inner (10–50%) and outer (50–90%) myocardium to form 32 subsegments [21]. A further circumferential subdivision of segments into 3 equiangular (sub-)subsegments each resulted in a total of 96 (sub-)subsegments (Fig. 2). Subsequently, signal intensity (SI) time curves extraction and Fermi deconvolution analyses were used to translate the SI values into blood flow values for each (sub-)subsegment, using an in-house software (Mathworks, Natick, Massachusetts, USA, version R2010b on a 64bit PC) [23]. Blood flow for each (sub-)subsegment was expressed as a percentage, relative to the flow value of the (sub-)subsegment with the highest flow (defining the normal reference of myocardial perfusion intra-individually), i.e. the AHA segment with the highest flow in the 16 segment analysis (#16), the subsegment with the highest flow in the 32 subsegment analysis (#32), and the (sub-)subsegment with the highest flow in the 96 (sub-)subsegment analysis (#96). Additional analysis was performed for 48 transmural segments (#48). The (sub-)subsegment with the lowest flow per subject was used to represent the subject in the receiver operating characteristics (ROC) curve analyses for each segmentation. Also, the endo−/epicardial myocardial flow ratios were calculated for a given pair within a transmural segment, resulting in 16 and 48 ratios for the 32 and 96 segmentation models, respectively. Data was normalised to the highest ratio, i.e. the most normal pair of endo−/epicardial segments, and expressed as a percentage relative to this value. The lowest relative ratio per subject was used to represent this subject in the ROC curve analyses. Additional analyses were performed by employing a cut-off for abnormal blood flow, based on the quantification of myocardial perfusion in control patients (defined as mean – 2 standard deviations) and the number of (sub-)segments below the threshold for patients with no CAD and single, dual, and triple vessel disease. The results are provided in % myocardium as a measure for the total ischaemic burden.

### Statistical analysis

Statistical analysis was performed using the SPSS (version 25.0). Departures from normality were examined using Shapiro-Wilk’s test. Data is presented in counts (percentages), mean ± standard deviation (SD), or median (interquartile range, IQR), as appropriate for the type of the data. Comparisons of means were performed using independent samples, t-test or one-way Analysis of Variance (ANOVA), Chi^−^squared, and Mann-Whitney test, as appropriate. Fischer’s exact tests were employed for proportions. ROC curve analyses were used to test the ability of CMR measures to discriminate between the groups. Reproducibility of postprocessing approaches were assessed using Bland-Altman analyses. All tests were two-tailed and a *p*-value of < 0.05 was considered statistically significant.

## Results

Characteristics of the study population are summarised in Table 1. Patients and controls were similar for age and gender. Angina was the most common presenting symptom, followed by dyspnoea, and arrhythmic presentations. Compared to controls, CAD patients had significant higher blood pressure, cardiac volumes and LV mass (*p* < 0.01 for all). Interestingly, LV ejection fraction (LVEF) remained preserved in an increasing percentage of patients with CAD due to highly effective and rapid therapeutic strategies. A majority of the CAD patients took a number of cardiac medications, including statin and anti-anginal therapy. Fifty-three percent of patients underwent previous percutaneous coronary intervention (*n* = 16, 53%),

**Table 1:** Subjects’ characteristics, medication and cardiovascular magnetic resonance (CMR) findings

Variables	Controls (*n* = 20)	CAD patients (*n* = 30)	Significance (*p*-value)
Age (years)	49 ± 13	53 ± 12	0.27
Males, n (%)	12 (60)	16 (53)	0.631
BMI, kg/m^2^	26 ± 3	28 ± 4	0.063
Heart rate, bpm	61 ± 12	60 ± 11	0.763
BP systolic, mmHg	115 ± 9	132 ± 11	0.304
BP diastolic, mmHg	72 ± 10	76 ± 11	0.198
Hypertension, n (%)		24 (80)	
Diabetes mellitus (type 2), n (%)		17 (57)	
Hypercholesterolemia, n (%)		28 (93)	
Smoking, n (%)		16 (53)	
Angina CCS > II, n (%)		18 (60)	
Dyspnoea, n (%)		12 (40)	
History of PCI n (%)		16 (53)	
Cardiac medication			
Aspirin, n (%)		27 (90)	
Anticoagulation, n (%)		3 (10)	
Betablockers, n (%)		26 (87)	
Calcium channel blockers, n (%)		17 (57)	
RAS-Inhibitors, n (%)		26 (87)	
Lipid-lowering therapy, n (%)		27 (90)	
Antianginals, n (%)		16 (53)	
Blood markers			
Haematocrit (%)	44 ± 4	42 ± 6	0.197
eGFR, ml/min/1.73 m2	84 ± 5	69 ± 8	< 0.001
Hs-CRP, mg/l	3.6 ± 2.8	4.8 ± 3.3	0.006
Cath findings			
Single vessel disease n (%)		14 (47)	
LAD		6	
RCX		3	
RCA		6	
3-vessel-disease or equivalent, n (%)		6 (20)	
CMR measures of function and structure			
LV-EDV (index), ml/m^2^	78 ± 9	81 ± 8	0.223
LV-ESV (index), ml/m^2^	32 ± 8	34 ± 7	0.355
LV-EF, %	61 ± 5	59 ± 6	0.224
LV-mass (index), g/m^2^	58 ± 8	74 ± 7	< 0.001
RV-EF, %	54 ± 6	56 ± 6	0.254
Myocardial LGE, present, n (%)	/	12 (40)	/

*BP* Blood pressure, *BMI* Body mass index, *CAD* Coronary artery disease, *RAS* Renin angiotensin system, *eGFR* Estimated glomerular filtration rate, *hs-CRP* High sensitive C-reactive protein, *LV* Left ventricular, *EDV* End-diastolic volume, *ESV* End-systolic volume, *EF* Ejection fraction, *LA* Left atrium, *LG*E Late gadolinium enhancement, *PCI* Percutaneous coronary intervention, *RCA* Right coronary artery, *LAD* Left anterior descending coronary artery, *LCX* Left circumflex coronary artery, *RV* Right ventricle/ventricular

**Fig. 3:**
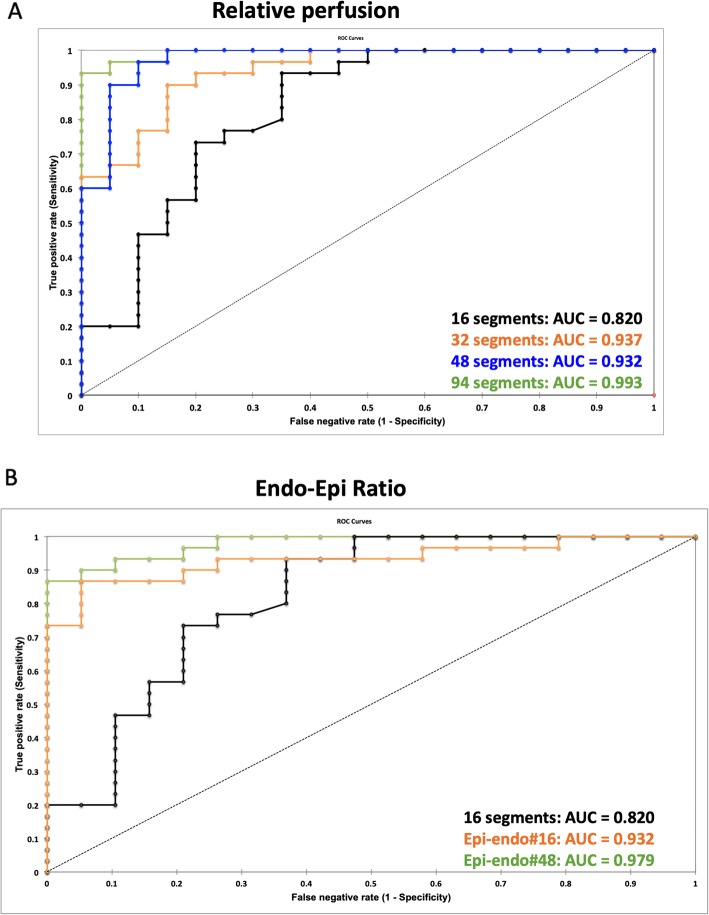
Results of receiver operator characteristics (ROC) curve analysis in identifying subjects with significant coronary artery disease (CAD). **a**: ROC curves for 16 segments (black), 32 segments (orange) and 96 segments (green); **b**: ROC curves for 16 segments (black), endo−/epicardial ratio based on 32 segments (orange) and endo−/epicardial ratio based on 96 segments (green)

**Fig. 4:**
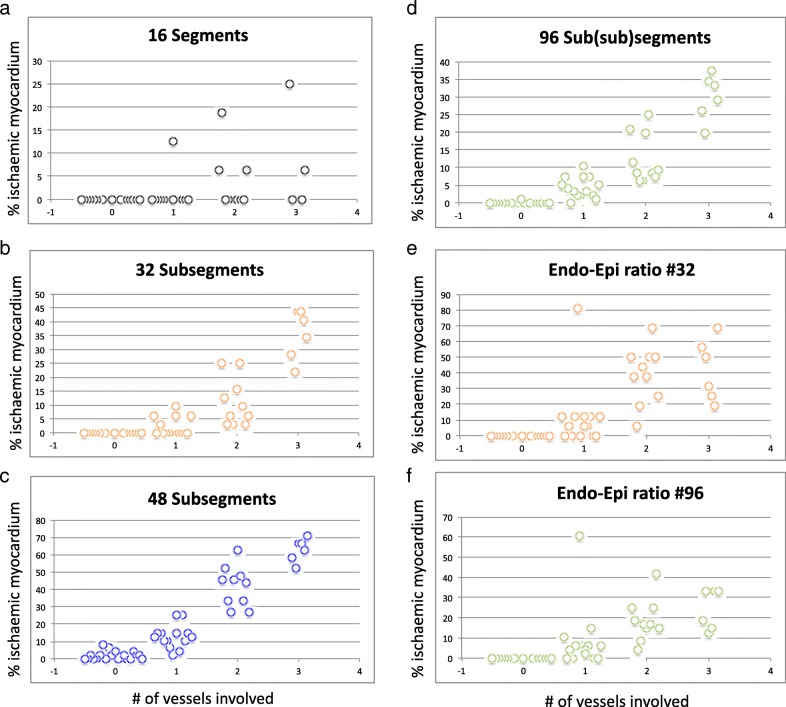
Scatterplots demonstrating percent ischaemia in controls vs. CAD patients. For controls, single vessel disease (1-VD), dual vessel disease (2-VD) and triple vessel disease (3-VD) for the classic 16 segment analysis (**a**), 32 subsegment analysis (**b**) 48 subsegment analysis (**c**) 96 subsegment analysis (**d**), endo−/epicardial ratio based on 32 segments (Endo-Epi ratio #16) (**e**) as well as endo-epicardial ratio based on 96 segments (Endo-Epi ratio #48) (**f**)

The results of the ROC curve analyses for agreement between relative peak perfusion and subjects group allocation are presented in Figs. 3a and b and Table 2. Results with more numerous and smaller segments revealed greater accuracy for #32, #48 and # 96 (area under the curve (AUC): 0.937, 0.973 and 0.993, *p* < 0.001), when compared to the standard #16 approach (AUC: 0.820, p < 0.001), and the superiority of #96 when compared to #48, #32, and #16 segment model (Table 2, *p* < 0.05 for all). Furthermore, the endo−/epicardial ratio, based on 96 segments (endo−/epicardial#48, AUC: 0.979, p < 0.001), was significantly superior to the endo−/epicardial ratio, based on 32 segments (endo−/epicardial#16, AUC 0.932, p < 0.001), the transmural 16 segment model, and the 48 segment model in identifying subjects with obstructive CAD (p < 0.05). Compared to controls, patients with more severe CAD had more ischaemic segments in any of the segmentation models, however, the threshold for abnormality was rarely met for the 16-segment model. The number of (sub-)segments below the mean - 2 SD is represented in Fig. 4 a-d. The endo−/epicardial ratio demonstrated a high number of positive segments in all patients but suffered from outliers in patients with no coronary disease, as well as single or dual vessel disease (in Fig. 4 e-f).

**Table 2:** Results of receiver operating characteristics curve analyses and comparisons

	Area under the curve (95% Confidence Interval)	Significance (*p*-value)	Sensitivity	Specificity	Area under the curve comparisons			
					Z	*p*-value	z	*p*-value
#16	0.820 (0.690–0.951)	< 0.001	0.93	0.63	Vs. 16		Vs. 32	
#32	0.937 (0.875–0.998)	< 0.001	0.90	0.84	2.15	0.032		
#48	0.973 (0.933–1.00)	< 0.001	0.97	0.90	1.96	0.015	1.38	0.167
#96	0.993 (0.981–1.00)	< 0.001	0.93	1.00	2.67	0.008	2.02	0.044
endo−/epicadial#16	0.932 (0.862–1.00)	< 0.001	0.87	0.95	1.70	0.89		
endo−/epicardial#48	0.979 (0.952–1.00)	< 0.001	0.87	1.00	2.57	0.015	1.82	0.069

### Reproducibility

Bland-Altman graphs are provided in the **supplementary material** and demonstrate marginally, but not significantly, better reproducibility of measurements for the #16 approach (mean difference ± standard deviation (MD ± SD): 2.0 ± 3.62) when compared to the #32 approach (MD ± SD: 2.46 ± 4.37), the #48 approach (MD ± SD: 2.32 ± 4.03) and the #96 approach (MD ± SD: 4.1 ± 5.62), as well as for the epi- and endocardial #16 approach (MD ± SD: 4.37 ± 5.91) vs. the endo- and epicardial #48 approach (MD ± SD: 7.97 ± 9.21) in Additional file 1: Fig. S1A-E**).**

## Discussion

In the current analysis we demonstrate that further segmentation of the classical AHA 16 segment model into smaller segments for the quantification of myocardial perfusion CMR improves diagnostic accuracy at the expense of lower intra-observer reproducibility. The highest agreement with CAD was obtained with (sub-)subsegmentation of LV in a total of 96 subsegments (#96), which was achieved by a division of the 16 AHA segments into an epi- and an endocardial layer (#32) with further subdivision into 3 equiangular subsegments.

Current clinical standard reporting is based on a visual impression of discerning the area of hypoperfusion relative to other segments. This area is visually graded for size and localisation and assigned to a coronary artery territory. Whereas such report is primarily descriptive, it is effective in offering most of the clinically relevant information to the referring clinician, conferred with high positive and negative predictive value of identifying prognostically relevant CAD [24, 25]. The exact definitions of the positivity of a segment are lacking due to several reasons: perfusion defects frequently stretch beyond the border zone of a single segment. While maintaining the relationship with vessel territory is less of an issue for visual clinical reporting, the problems occur for databasing since clear “yes” or” no” decisions for each segment result in an overestimation of the defects (if the observer classifies any segment with a partial perfusion defect as positive), an underestimation of the defect (if the observer classifies only fully ischaemic segments as positive), and, consequently, a low reproducibility, if no clear rule is defined. A resulting disadvantage of the crude 16 segment model is the difficulty of concurring information of the extent of ischaemia between the visual impression and the segmental report due to the above-mentioned differences in interpretation. It is increasingly recognised that the severity of ischaemia, determined by the percentage of affected myocardium (e.g. > 10% ischaemic myocardium), is more important that its pure presence, making the extent of ischaemia an important CMR endpoint for clinical studies [4]. While a visual assessment accounts for the partially ischaemic segments in clinical interpretation, databasing offers no such solution. Consequently, current descriptive reports and the 16 segment-based databases have severe limitations in extracting relevant information about the extent or severity of ischaemia, making it difficult or impossible to accurately interpret outcome studies or to compare results using various definitions. This problem is even more pronounced on an inter-modal basis [10].

Recent developments of automated analyses [26], as well as the advances in algorithms supporting machine learning, enable analysis of small areas of myocardial tissue or even voxel-wise analyses.

We systematically compared the use of the 16 segments standard to a finer scale of up to 96 (sub-)subsegments. In addition, we assessed the performance of the endo−/epicardial gradient for 16 and 48 segments. The fundamental principle behind our concept is reducing the mixing of the noise and signal by avoiding the transmural segmental averaging – i.e. not using the mean of the segment. Given that a high ratio is regarded as normal and a low ratio demonstrates the occurrence of myocardial ischemia – as the endocardium has a lower perfusion pressure and a higher resistance due to intracavity LV pressure – we considered the segment with the highest SI as the most normal segment and the lowest SI as the most abnormal segment describing ischemia, with a dynamic range of values between these two designations. This allows to delineate the contrast between such segments and assign them as different. The capillary bed is indeed different between the epi- and the endocardium - a fact frequently neglected in perfusion analysis - and any segmental model with finer granularity will start catching these differences.

Using smaller segments creates numerous advantages and disadvantages.

Advantages:

i) Smaller segments improve the diagnostic accuracy of quantitative perfusion analysis. This is primarily due to a larger difference between normal and abnormal segments, as smaller segments have a higher likelihood of being fully normal or fully abnormal.
ii) Smaller segments allow for a better description of the extent of the ischaemic area based on objective cut-off values. Again, this is due to the larger difference between normal and abnormal segments. Using the 16-segment model, only the most severely affected segments reach a threshold defined as 2 standard deviations below the mean of the control group.

Disadvantages:

i) The utility of quantitative approaches in clinical management beyond the visual assessment remains widely debated. Currently, an approximation of 10% of total myocardium is being made by a division of the number of affected segments and the total number of segments. Yet, the interventional cardiologists continue to rely on the binary information (ischaemia yes/no) and the localisation in its relation to a major coronary artery. Smaller segments and better descriptions of the ischaemic areas may result in a greater precision of a clinically relevant threshold, allowing for a greater array of optimised treatment actions.
ii) Precise databasing, i.e. collection of data, is the most relevant first step towards generating such evidence. Smaller segments will create significant additional information and postprocessing workload, which may not be practical unless automated postprocessing methods are used. Documentation requires a more sophisticated database, which is harder to read for human observers.
iii) Increased subdivision of segments will increase the heterogeneity of SI values, as averaging across smaller and smaller segments will tease out a greater SI difference between segments with normal perfusion and those that are hypoperfused. This will also result in greater spread (or dispersion) of values, describing the myocardial perfusion. A smaller averaging area will inevitably lead to a stronger effect of outliers or inaccuracies in border delineation, resulting in greater observer variability, as seen in the present study. Notably, despite this limitation, the diagnostic accuracy of (sub-)subsegmentation led to significantly improved diagnostic accuracy. Together, this observation mandates further improvement of image acquisition (i.e. it cannot be solved solely by postprocessing), as it can possibly be resolved by more robust sequences with higher spatial resolution, reducing the dark rim artefacts, and fully automated postprocessing methods [12], [26, 27]

We regard catching these differences a strength (as shown by the data) rather than a weakness. However, once a resolution on a micro-meter scale is achievable these inhomogeneities may cause novel challenges / opportunities.

Interestingly, LVEF of both groups was not dissimilar. This observation resonates with our recent findings showing that in current CAD patients, the reduced LVEF has reduced prognostic power, unless in the presence of significant amount of scar [15]. With current treatment approaches in CAD, the LVEF is increasingly less profoundly affected, reflecting the success of the guidelines directed therapy in reducing the infarction scar and the postinfarction remodelling.

### Limitations

This is a proof-of concept study of a novel approach, and as such, is geared to inform on the effect size and not in a possession of one a priori. Previous studies in myocardial perfusion quantification have served as a guidance on the likely numbers required [4]. The present results were obtained in a training dataset from a relatively small sample of selected patients, thus, the cut-offs and the diagnostic accuracy cannot be immediately transferred to the general population, before the results are validated by a larger clinical population. Additionally, the sample was too limited to inform on potential age- or gender- related issues. Thus, the diagnostic accuracy and the cut-off values may not be transferable. However, the data demonstrate the statistically significant superiority of (sub-)subsegmentation.

No full quantification in mg blood flow per gram tissue per minute was performed. Firstly, the transfer of SI time curves to absolute flow requires models with a considerable amount of assumptions, which may not be fulfilled in perfusion CMR. Secondly, an advantage of the current method is the calculation of relative values to peak perfused areas. This approach “autocorrects” for issues such as the nonlinearity of an input function. Relying on the relative maximal SI difference between subsegments within a single acquisition means that a stress-only examination can be used. This is important, because current datasets were obtained in a registry of clinically indicated CMR studies, where rest perfusion is not performed routinely in order to minimise the total amount of gadolinium contrast agent dose, reducing the duration and the cost of the examination [28]. Comparative studies with quantification approaches that necessitate rest perfusion may be needed.

## Conclusions

Subsegmentation of the myocardium improves diagnostic accuracy and facilitates an objective cut-off-based description of hypoperfusion, and thus, the extent and severity of myocardial ischaemia. Quantification based on a single (stress-only) pass reduces the overall amount of gadolinium contrast agent required and the length of the overall diagnostic study.

## Supplementary information

**Additional file 1: Figure S1.** Bland-Altman plots for 16 segment analysis (A), 32 segment analysis (B), 96 segment analysis (C), endo−/epicardial ratio based on 32 segments (D) and endo−/epicardial ratio based on 96 segments (E).

## Data Availability

All data generated and analysed during this study are included in this published article.

## Abbreviations

1-VD: One vessel disease
2-VD: Two vessel disease
3-VD: Three vessel disease
AHA: American heart association
ANOVA: Analysis of variance
AUC: Area under the curve
BP: Blood pressure
CAD: Coronary artery disease
CMR: Cardiovascular magnetic resonance
FFR: Fractional flow reserve
GBCA: Gadolinium-based contrast agent
IQR: Interquartile range
LGE: Late gadolinium enhancement
LV: Left ventricle/left ventricular
LVEF: Left ventricular ejection fraction
MD: Mean difference
ROC: Receiver operator characteristics
SD: Standard deviation
SI: Signal intensity
