# Supplementary material for: Sub-segmental quantification of single (stress)-pass perfusion CMR improves the diagnostic accuracy for detection of obstructive coronary artery disease
Source: J Cardiovasc Magn Reson. 2020 Feb 6;22:14. doi: 10.1186/s12968-020-0600-1 (PMC7006214; doi:10.1186/s12968-020-0600-1)

**Supplementary material**

Perfusion quantification analysis

The complete method description has been published previously (1). In brief, motion was minimized by individually adapted breath-holding in expiration during the first pass of the contrast agent, inline motion correction using affine image registration (2) and a temporal maximum intensity projection serving as a feature image for automatic contour delineation based on active contours (3,4). Manual correction was applied when required to avoid blood-pool or para-cardiac structures. Voxel-wise signal intensities were then sampled using MEDIS Suite (Medis, Leiden, The Netherlands) and divided into 48 equiangular segments. The transmural positions were located on chords perpendicular to the myocardial centreline and the inner and outer 10% of the data was discarded. The remaining segments were divided into endo- and epicardial subsegments of equal width. The arterial input function was obtained within a region of interest (ROI) in the blood pool of the basal slice carefully avoiding papillary muscles using a diluted prebolus (5%). Before deconvolution analysis, baseline correction that includes scaling of the signal intensities proportional to coil sensitivity and correcting for an offset to shift the baseline signal to zero was performed. Spatial filtering was performed with a gaussian filter of size 5, temporal filtering with a 30th order low pass Hamming filter (5). Perfusion estimates were then computed by deconvolving the measured blood and tissue enhancement data during the first pass of contrast agent in myocardium.

1. [Zarinabad N](https://www.ncbi.nlm.nih.gov/pubmed/?term=Zarinabad%20N%5BAuthor%5D&cauthor=true&cauthor_uid=22354744)1, [Chiribiri A](https://www.ncbi.nlm.nih.gov/pubmed/?term=Chiribiri%20A%5BAuthor%5D&cauthor=true&cauthor_uid=22354744), [Hautvast GL](https://www.ncbi.nlm.nih.gov/pubmed/?term=Hautvast%20GL%5BAuthor%5D&cauthor=true&cauthor_uid=22354744), [Ishida M](https://www.ncbi.nlm.nih.gov/pubmed/?term=Ishida%20M%5BAuthor%5D&cauthor=true&cauthor_uid=22354744), [Schuster A](https://www.ncbi.nlm.nih.gov/pubmed/?term=Schuster%20A%5BAuthor%5D&cauthor=true&cauthor_uid=22354744), [Cvetkovic Z](https://www.ncbi.nlm.nih.gov/pubmed/?term=Cvetkovic%20Z%5BAuthor%5D&cauthor=true&cauthor_uid=22354744), [Batchelor PG](https://www.ncbi.nlm.nih.gov/pubmed/?term=Batchelor%20PG%5BAuthor%5D&cauthor=true&cauthor_uid=22354744), [Nagel E](https://www.ncbi.nlm.nih.gov/pubmed/?term=Nagel%20E%5BAuthor%5D&cauthor=true&cauthor_uid=22354744). Voxel-wise quantification of myocardial perfusion by cardiac magnetic resonance. Feasibility and methods comparison. [Magn Reson Med.](https://www.ncbi.nlm.nih.gov/pubmed/22354744) 2012 Dec;68(6):1994-2004.
2. Xue H, Zuehlsdorff S, Kellman P, Arai A, Nielles-Vallespin S, Chefdhotel C, Lorenz CH, Guehring J. Unsupervised inline analysis of cardiac perfusion MRI. Med Image Comput Comput Assist Interv. 2009;12(Pt 2):741-9.
3. Breeuwer M, Quist M, Spreeuwers L.Automatic quantitative analysis of cardiac MR perfusion images. In: Proceedings of SPIE Medical Imaging, San Diego, CA, USA 2001. pp 733–742.
4. Spreeuwers L, Breeuwer M. Automatic detection of myocardial boundaries in MR cardio perfusion images. In: Proceedings of Medical Image Computing and Computer‐Assisted Intervention, Utrecht, Netherlands,2001. pp 1228–1231
5. Di Bella EV, Wu YJ, Alexander AL, Parker DL, Green D, McGann CJ. Comparison of temporal filtering methods for dynamic contrast MRI myocardial perfusion studies. Magn Reson Med. 2003; 49: 895—902

**Figures Suppl 1**: Bland-Altman plots for 16 segment analysis (A), 32 segment analysis (B), 48 segment analysis (C), 96 segment analysis (D) Endo-Epi ratio based on 32 segments (E) and Endo-Epi ratio based on 96 segments (F).

**Fig 1A**
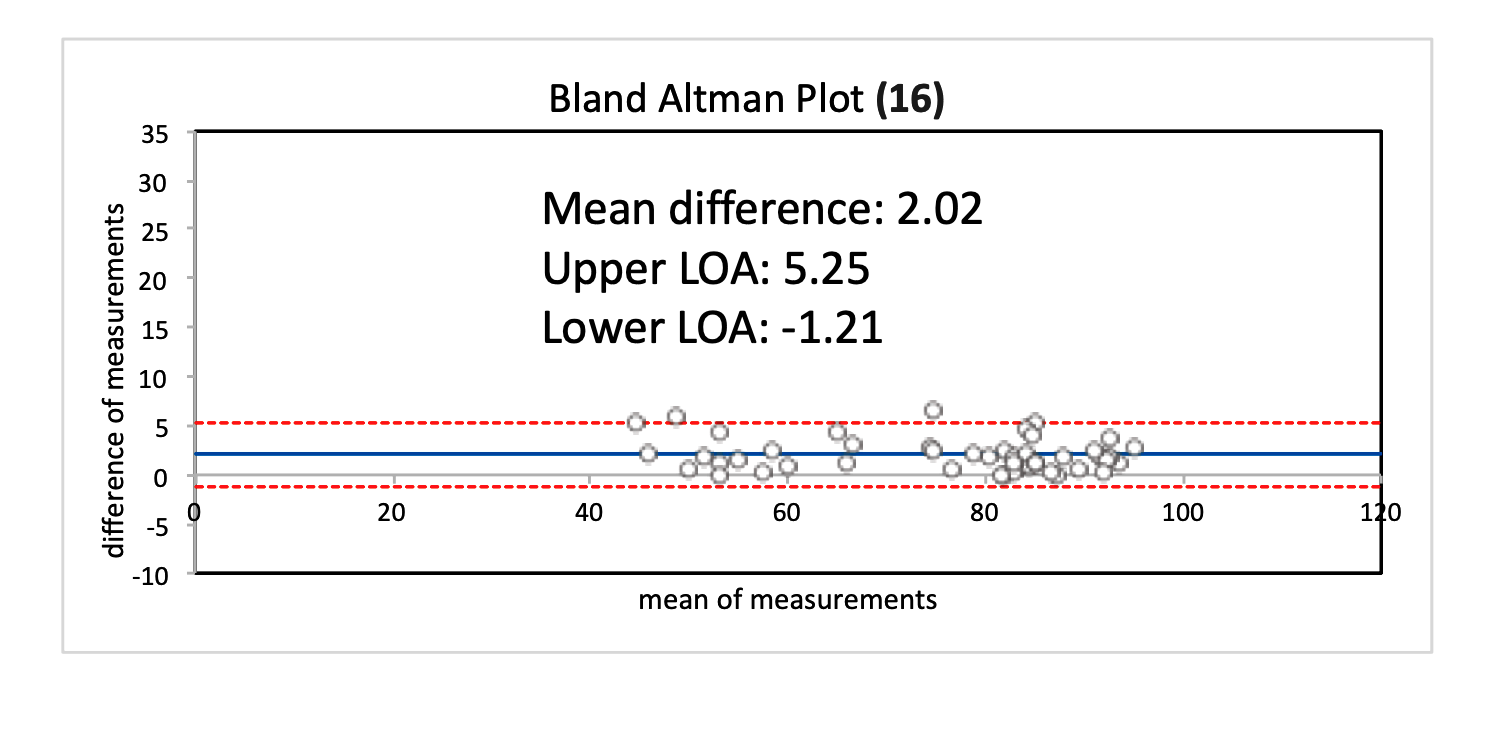


**Fig 1B**
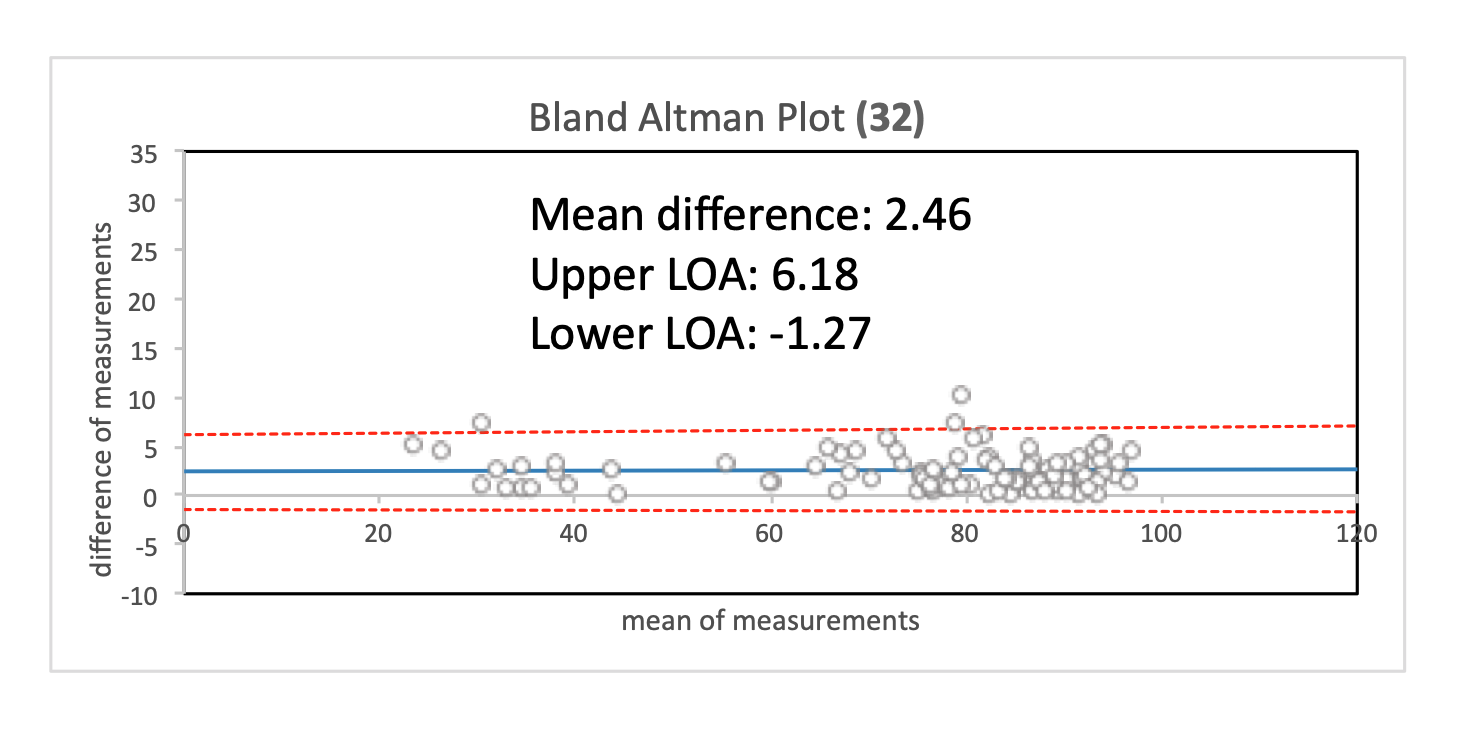


**Fig 1C**
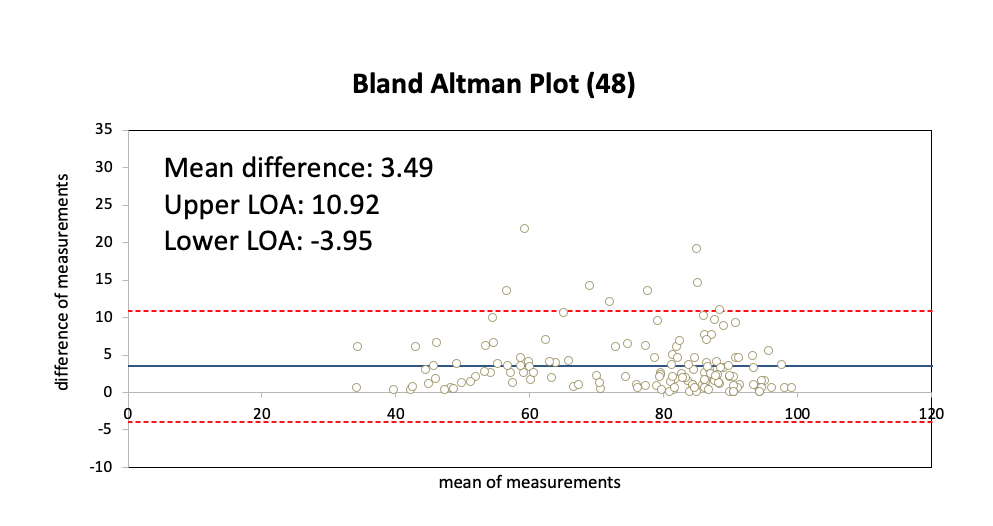


**Fig 1D**


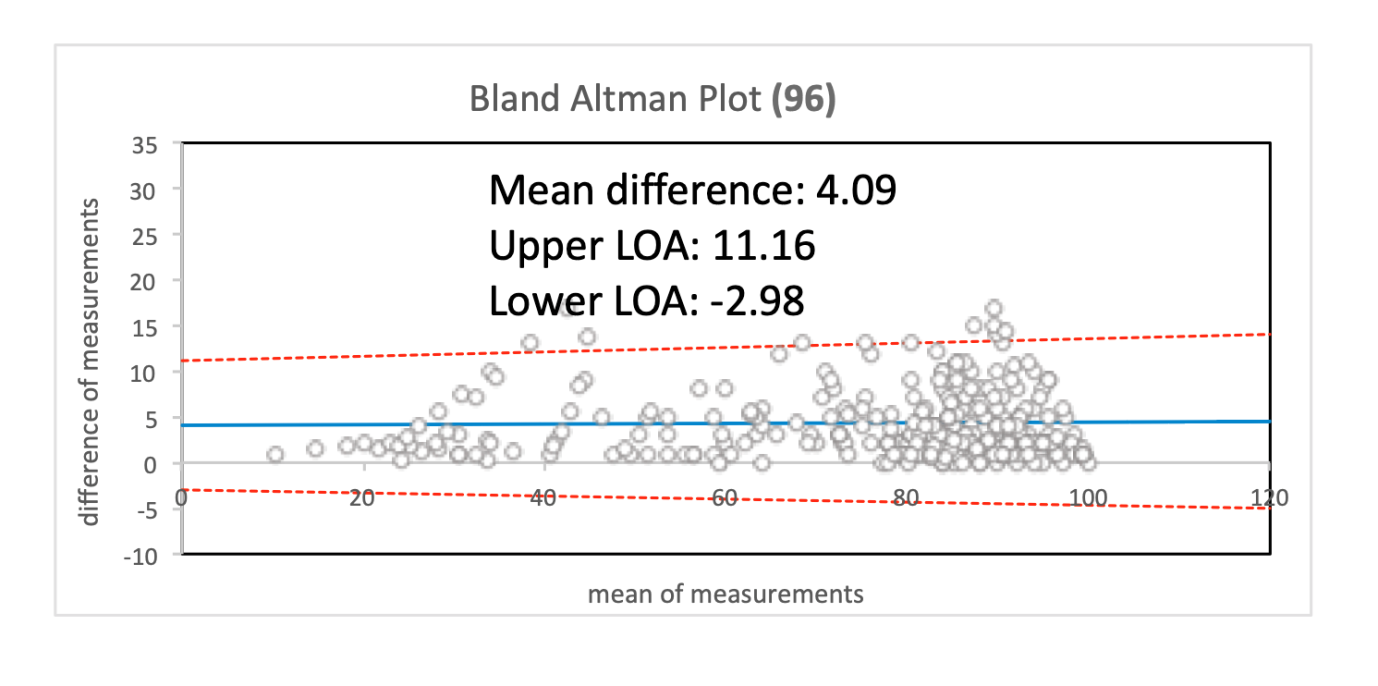


**Fig 1E**


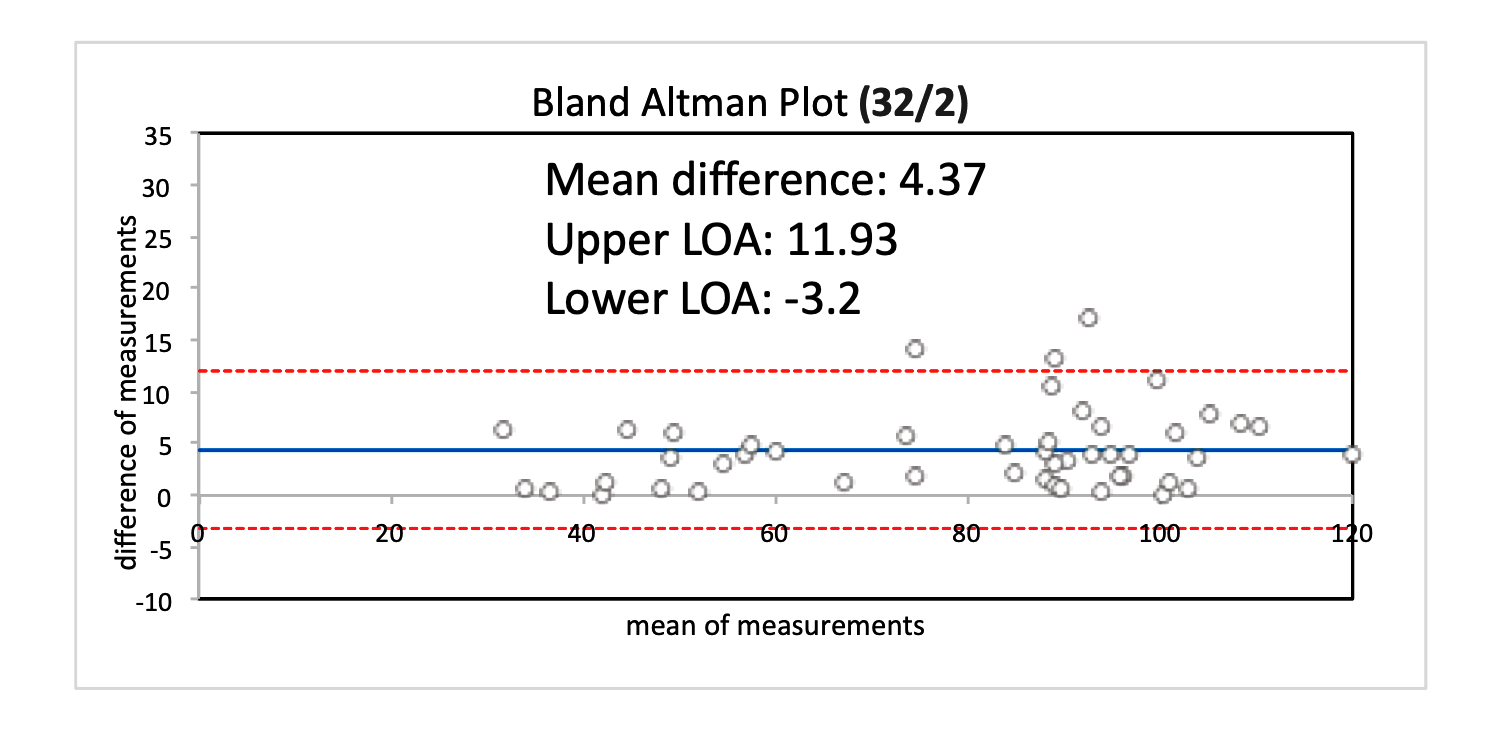
 **Fig 1F**
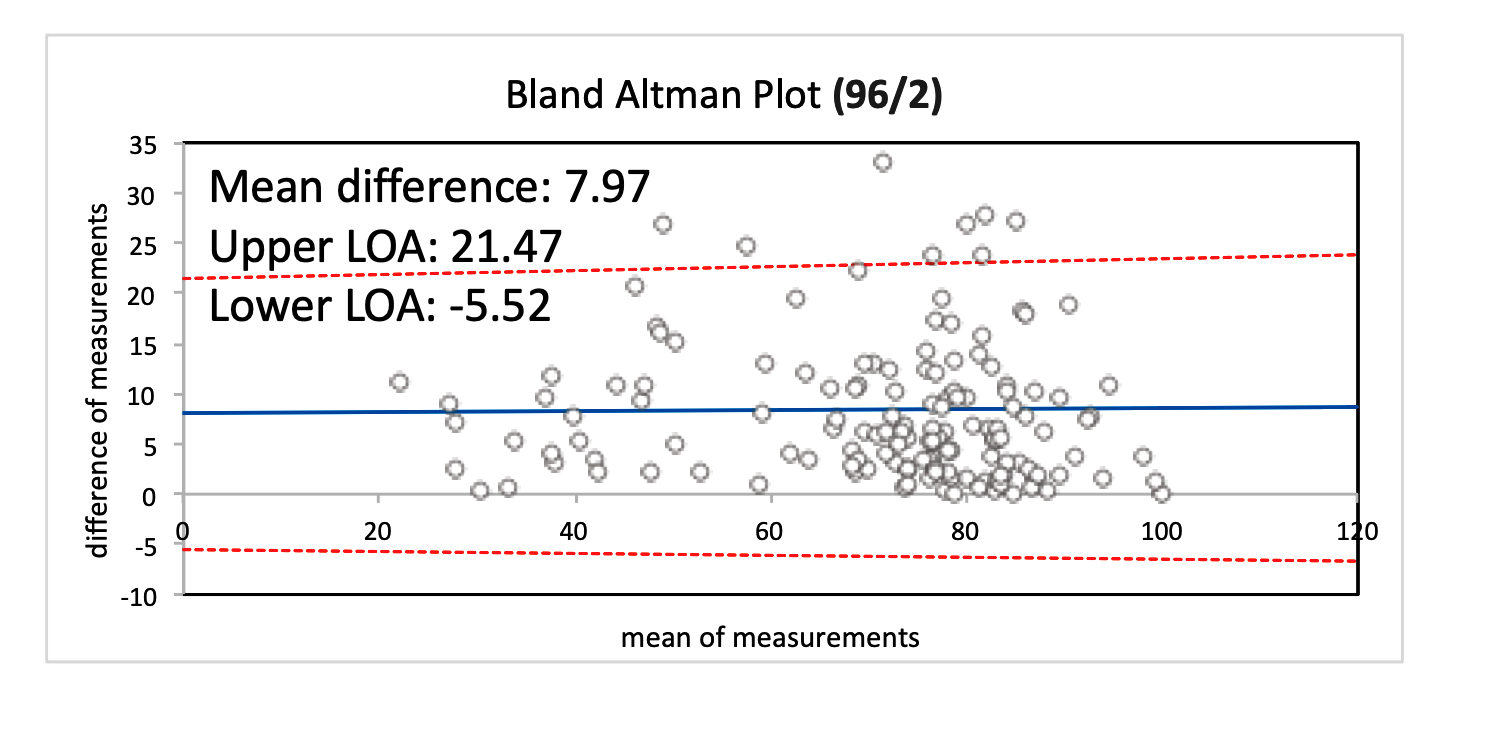

Supplement: Supplementary file 1 — Additional file 1: Figure S1. Bland-Altman plots for 16 segment analysis (A), 32 segment analysis (B), 96 segment analysis (C), endo−/epicardial ratio based on 32 segments (D) and endo−/epicardial ratio based on 96 segments (E). [file 12968_2020_600_MOESM1_ESM.docx]
